# Action of Mercury on the Secretion of Bile

**Published:** 1859-10

**Authors:** 


*Action of Mercury on the Secretion of Bile.*—Among the effects of medicine which are usually considered to be well established, the action of mercurial preparations in increasing the flow of bile has been admitted without question. Physicians speak about stimulating a sluggish liver by blue pill, or calomel, as if it were not only the easiest thing in the world, but as if we had ocular demonstration of the process; and the frequency with which the liver is thus stimulated to an increased secretion of bile is in proportion to the ease with which it is supposed this may be effected. No matter what the disease, it is the commonest thing to begin the treatment by an active mercurial purgative.

Some experiments by Dr. George Scott, of Southampton, Eng., made on dogs, with a view of ascertaining whether mercury really increases the flow of bile, lead to the conclusion that the hitherto-received opinions on this subject are erroneous, and that calomel at least does not increase the biliary secretion. Having ascertained the average quantity of bile secreted in twenty-four hours, by collecting it in a vessel, after the common duct was tied, Dr. Scott administered calomel to the dogs, and then noted the amount of bile, the quantity of food and drink taken being the same. The four experiments of Dr. Scott all gave the same result,—that *there was a diminution in the amount of bile secreted after the administration of large doses of calomel.* If these experiments should be confirmed by future ones, a revolution may be expected in the treatment of diseases supposed to be connected with a deficiency in the biliary secretion; and that much-abused organ, the liver, will be allowed some rest from the incessant appeals which are made to it as the source of so many functional diseases. We are glad that Dr. Scott has undertaken to investigate the effects of calomel on the liver by direct experiment. We hope he will continue his researches, and extend them to other subjects connected with the action of medicines. There is no department of our science in which so little is known, or in which more light seems capable of being thrown by direct experiment.—*The Boston Medical and Surgical Journal.*

